# Supplementary material for: Methylglyoxal, Glycated Albumin, PAF, and TNF-α: Possible Inflammatory and Metabolic Biomarkers for Management of Gestational Diabetes
Source: Nutrients. 2020 Feb 14;12(2):479. doi: 10.3390/nu12020479 (PMC7071306; doi:10.3390/nu12020479)
Supplement: Supplementary file 1 [file nutrients-12-00479-s001.pdf]

Table S1. Table of correlation coefficients for all biomarker variables at each time point.

	Age	Pre-gestational weight	Pre-gestational BMI	Birth weight	Fasting blood glucose T0
**Age**		0.712	0.671	0.597	0.349
**Pre-gestational weight**	-0.070		0.000	0.003	0.195
**Pre-gestational BMI**	-0.081	0.863		0.031	0.260
**Birth weight**	0.114	0.580	0.441		0.799
**Fasting blood glucose T0**	-0.196	0.268	0.234	0.059	
**Glycated hemoglobin T0**	0.090	0.141	0.209	-0.015	0.479
**Insulin T0**	0.150	0.193	0.279	0.082	0.012
**HOMA index T0**	-0.136	0.431	0.493	-0.024	0.331
**Total cholesterol T0**	-0.232	-0.072	-0.099	-0.010	0.002
**LDL cholesterol T0**	-0.265	-0.275	-0.237	-0.134	-0.028
**HDL cholesterol T0**	-0.068	0.156	-0.031	0.378	-0.173
**Triglycerides T0**	0.025	0.198	0.195	0.183	0.012
**CRP T0**	-0.119	0.023	0.168	0.053	-0.065
**Cortisol T0**	-0.107	-0.298	-0.211	-0.225	-0.097
**BAFF T0**	-0.205	-0.268	-0.120	-0.280	0.050
**PAF T0**	-0.049	0.068	0.112	-0.293	0.259
**TNF-α T0**	0.033	-0.047	0.012	0.010	0.049
**MG T0**	-0.190	0.400	0.272	0.438	0.079
**GA T0**	-0.226	0.388	0.417	0.280	-0.013
**glic/tot T0**	-0.221	0.372	0.364	0.348	-0.168
**Fasting blood glucose T12**	-0.372	0.055	0.010	0.139	0.446
**Glycated hemoglobin T12**	0.033	0.071	0.183	0.049	0.580
**Insulin T12**	0.012	0.051	0.130	-0.386	0.296
**HOMA index T12**	-0.283	0.147	0.312	-0.318	0.316
**Total cholesterol T12**	-0.080	-0.378	-0.330	-0.105	-0.209
**LDL cholesterol T12**	-0.050	-0.293	-0.229	0.019	-0.099
**HDL cholesterol T12**	-0.093	0.027	-0.088	0.222	-0.082
**Triglycerides T12**	0.161	0.226	0.271	0.299	0.056
**CRP T12**	-0.119	0.069	0.034	0.129	0.031
**Cortisol T12**	0.276	-0.307	-0.257	-0.313	-0.033
**BAFF T12**	-0.067	0.100	0.266	0.045	0.216
**PAF T12**	0.170	0.160	0.171	-0.092	0.150
**TNF-α T12**	0.163	0.240	0.296	0.114	0.218
**MG T12**	-0.264	0.406	0.301	0.397	0.178
**GA T12**	-0.064	0.229	-0.020	0.208	0.019
**glic/tot T12**	-0.181	0.399	0.202	0.372	-0.163

	Glycated hemoglobin T0	Insulin T0	HOMA index T0	Total cholesterol T0	LDL cholesterol T0
**Age**	0.635	0.430	0.527	0.218	0.156
**Pre-gestational weight**	0.457	0.307	0.036	0.704	0.141
**Pre-gestational BMI**	0.269	0.135	0.014	0.601	0.207
**Birth weight**	0.945	0.702	0.920	0.963	0.534
**Fasting blood glucose T0**	0.016	0.953	0.115	0.994	0.893
**Glycated hemoglobin T0**		0.024	0.096	0.910	0.927
**Insulin T0**	0.412		0.000	0.482	0.709
**HOMA index T0**	0.348	0.970		0.224	0.558
**Total cholesterol T0**	-0.022	0.133	0.258		0.000
**LDL cholesterol T0**	-0.017	0.071	0.126	0.892	
**HDL cholesterol T0**	-0.414	-0.250	-0.178	0.433	0.197
**Triglycerides T0**	0.231	0.225	0.298	0.253	0.091
**CRP T0**	0.115	0.329	0.533	0.337	0.361
**Cortisol T0**	-0.173	-0.264	-0.286	0.250	0.354
**BAFF T0**	-0.049	0.032	-0.004	0.239	0.300
**PAF T0**	0.394	0.197	0.479	-0.058	-0.034
**TNF-α T0**	-0.173	0.072	0.164	0.084	0.081
**MG T0**	0.401	0.338	0.440	0.330	0.283
**GA T0**	-0.207	-0.043	0.140	0.156	-0.030
**glic/tot T0**	-0.204	-0.005	0.056	0.149	-0.025
**Fasting blood glucose T12**	0.161	0.100	0.287	-0.098	-0.181
**Glycated hemoglobin T12**	0.746	0.322	0.289	0.086	0.107
**Insulin T12**	0.384	0.486	0.774	0.107	-0.017
**HOMA index T12**	0.254	0.400	0.768	0.304	0.247
**Total cholesterol T12**	-0.146	0.029	-0.106	0.822	0.821
**LDL cholesterol T12**	0.014	0.149	0.000	0.758	0.872
**HDL cholesterol T12**	-0.462	-0.088	-0.121	0.271	0.167
**Triglycerides T12**	0.038	0.006	0.017	0.094	-0.071
**CRP T12**	-0.137	-0.179	-0.078	0.282	0.186
**Cortisol T12**	-0.010	-0.391	-0.447	-0.136	0.075
**BAFF T12**	0.047	0.020	-0.082	0.132	0.100
**PAF T12**	0.364	0.291	0.422	-0.072	-0.118
**TNF-α T12**	-0.076	0.032	0.119	0.172	0.102
**MG T12**	0.458	0.273	0.220	0.390	0.319
**GA T12**	-0.538	-0.388	-0.164	0.112	0.083
**glic/tot T12**	-0.442	-0.178	-0.135	0.120	0.017

	HDL cholesterol T0	Triglycerides T0	CRP T0	Cortisol T0	BAFF T0
**Age**	0.720	0.894	0.532	0.572	0.276
**Pre-gestational weight**	0.412	0.294	0.902	0.110	0.152
**Pre-gestational BMI**	0.873	0.302	0.376	0.262	0.528
**Birth weight**	0.069	0.391	0.805	0.290	0.185
**Fasting blood glucose T0**	0.407	0.955	0.758	0.644	0.811
**Glycated hemoglobin T0**	0.023	0.219	0.546	0.362	0.797
**Insulin T0**	0.182	0.232	0.076	0.158	0.868
**HOMA index T0**	0.404	0.157	0.007	0.176	0.986
**Total cholesterol T0**	0.017	0.177	0.068	0.182	0.204
**LDL cholesterol T0**	0.297	0.634	0.050	0.055	0.107
**HDL cholesterol T0**		0.145	0.260	0.050	0.497
**Triglycerides T0**	-0.272		0.005	0.002	0.097
**CRP T0**	-0.212	0.502		0.140	0.381
**Cortisol T0**	0.361	-0.536	-0.276		0.931
**BAFF T0**	-0.129	0.309	0.166	0.016	
**PAF T0**	-0.498	0.344	0.327	-0.360	0.094
**TNF-α T0**	-0.261	0.182	0.205	-0.296	-0.097
**MG T0**	0.016	0.336	0.292	-0.015	0.075
**GA T0**	0.389	0.091	0.198	0.052	0.102
**glic/tot T0**	0.342	0.183	0.190	0.006	0.150
**Fasting blood glucose T12**	-0.299	0.294	0.326	-0.272	-0.015
**Glycated hemoglobin T12**	-0.355	0.272	0.078	-0.149	0.119
**Insulin T12**	-0.387	0.452	0.157	-0.284	0.307
**HOMA index T12**	-0.204	0.426	0.497	0.027	0.357
**Total cholesterol T12**	0.392	-0.014	0.252	0.397	0.176
**LDL cholesterol T12**	0.217	-0.076	0.342	0.414	0.123
**HDL cholesterol T12**	0.815	-0.563	-0.228	0.384	-0.105
**Triglycerides T12**	-0.126	0.730	0.153	-0.458	0.152
**CRP T12**	0.125	0.331	0.283	-0.373	0.161
**Cortisol T12**	0.099	-0.563	-0.473	0.647	0.185
**BAFF T12**	0.018	0.175	0.014	0.019	0.704
**PAF T12**	-0.368	0.350	0.182	-0.370	0.154
**TNF-α T12**	-0.148	0.277	0.254	-0.402	0.010
**MG T12**	0.170	0.156	0.033	0.118	0.125
**GA T12**	0.312	0.054	-0.058	-0.016	0.050
**glic/tot T12**	0.435	0.150	-0.026	-0.084	0.135

	PAF T0	TNF-α T0	MG T0	GA T0	glic/tot T0
Age	0.799	0.864	0.314	0.238	0.250
Pre-gestational weight	0.723	0.806	0.028	0.037	0.047
Pre-gestational BMI	0.555	0.948	0.146	0.024	0.052
Birth weight	0.165	0.965	0.032	0.196	0.104
Fasting blood glucose T0	0.211	0.815	0.708	0.953	0.434
Glycated hemoglobin T0	0.031	0.362	0.028	0.281	0.289
Insulin T0	0.296	0.707	0.067	0.825	0.978
HOMA index T0	0.018	0.443	0.031	0.524	0.800
Total cholesterol T0	0.762	0.658	0.075	0.420	0.442
LDL cholesterol T0	0.860	0.670	0.129	0.876	0.896
HDL cholesterol T0	0.005	0.164	0.933	0.037	0.069
Triglycerides T0	0.063	0.336	0.070	0.637	0.341
CRP T0	0.077	0.277	0.118	0.303	0.324
Cortisol T0	0.051	0.112	0.936	0.790	0.977
BAFF T0	0.620	0.610	0.695	0.599	0.436
PAF T0		0.818	0.708	0.690	0.610
TNF-α T0	0.044		0.321	0.237	0.086
MG T0	0.071	-0.188		0.717	0.898
GA T0	0.077	-0.227	-0.070		0.000
glic/tot T0	0.099	-0.325	0.025	0.940	
Fasting blood glucose T12	0.109	0.168	0.186	-0.032	-0.050
Glycated hemoglobin T12	0.295	0.124	0.074	-0.103	-0.170
Insulin T12	0.401	0.227	-0.028	0.051	-0.033
HOMA index T12	0.277	-0.056	0.233	0.296	0.197
Total cholesterol T12	-0.244	0.208	-0.009	0.029	-0.008
LDL cholesterol T12	-0.177	0.308	0.151	-0.031	-0.138
HDL cholesterol T12	-0.580	-0.288	-0.010	0.267	0.196
Triglycerides T12	0.250	0.348	-0.048	0.260	0.372
CRP T12	0.101	0.081	-0.086	0.090	0.015
Cortisol T12	-0.277	-0.434	-0.176	-0.243	-0.345
BAFF T12	-0.076	0.048	-0.125	0.234	0.208
PAF T12	0.756	-0.014	0.101	0.118	0.175
TNF-α T12	0.193	0.542	-0.241	0.211	0.148
MG T12	0.026	-0.331	0.824	-0.029	0.095
GA T12	0.102	-0.151	0.078	0.216	0.272
glic/tot T12	-0.014	-0.372	0.115	0.454	0.604

	Fasting blood glucose T12	Glycated hemoglobin T12	Insulin T12	HOMA index 12	Total cholesterol T12
**Age**	0.097	0.871	0.954	0.170	0.703
**Pre-gestational weight**	0.814	0.730	0.810	0.482	0.062
**Pre-gestational BMI**	0.965	0.370	0.537	0.129	0.107
**Birth weight**	0.571	0.821	0.069	0.185	0.634
**Fasting blood glucose T0**	0.049	0.005	0.192	0.142	0.362
**Glycated hemoglobin T0**	0.485	0.000	0.058	0.221	0.487
**Insulin T0**	0.667	0.108	0.014	0.047	0.890
**HOMA index T0**	0.233	0.203	0.000	0.000	0.656
**Total cholesterol T0**	0.674	0.678	0.612	0.139	0.000
**LDL cholesterol T0**	0.433	0.604	0.935	0.234	0.000
**HDL cholesterol T0**	0.188	0.075	0.056	0.329	0.053
**Triglycerides T0**	0.196	0.178	0.023	0.034	0.948
**CRP T0**	0.150	0.706	0.452	0.011	0.225
**Cortisol T0**	0.232	0.467	0.169	0.900	0.049
**BAFF T0**	0.950	0.563	0.136	0.080	0.400
**PAF T0**	0.637	0.143	0.047	0.181	0.240
**TNF-α T0**	0.466	0.545	0.275	0.789	0.319
**MG T0**	0.420	0.719	0.893	0.262	0.965
**GA T0**	0.895	0.625	0.812	0.160	0.894
**glic/tot T0**	0.834	0.416	0.879	0.356	0.969
**Fasting blood glucose T12**		0.536	0.408	0.083	0.131
**Glycated hemoglobin T12**	0.143		0.003	0.011	0.723
**Insulin T12**	0.191	0.563		0.000	0.791
**HOMA index T12**	0.387	0.541	0.959		0.579
**Total cholesterol T12**	-0.340	0.075	-0.056	-0.129	
**LDL cholesterol T12**	-0.276	0.216	-0.087	-0.027	0.942
**HDL cholesterol T12**	-0.263	-0.433	-0.488	-0.453	0.345
**Triglycerides T12**	0.034	0.339	0.316	0.196	-0.021
**CRP T12**	0.155	-0.005	0.074	0.118	0.253
**Cortisol T12**	-0.407	-0.123	-0.210	-0.190	0.111
**BAFF T12**	-0.126	0.449	0.272	0.259	0.150
**PAF T12**	0.096	0.357	0.521	0.237	-0.268
**TNF-α T12**	0.083	0.209	0.177	0.067	0.277
**MG T12**	-0.066	0.175	-0.142	0.091	0.131
**GA T12**	-0.067	-0.603	-0.442	-0.262	-0.015
**glic/tot T12**	-0.153	-0.458	-0.314	-0.165	-0.027

	LDL cholesterol T12	HDL cholesterol T12	Triglycerides T12	CRP T12	Cortisol T12
**Age**	0.822	0.659	0.441	0.571	0.182
**Pre-gestational weight**	0.175	0.899	0.277	0.742	0.136
**Pre-gestational BMI**	0.294	0.675	0.189	0.872	0.215
**Birth weight**	0.935	0.308	0.166	0.559	0.146
**Fasting blood glucose T0**	0.670	0.724	0.810	0.893	0.889
**Glycated hemoglobin T0**	0.949	0.020	0.856	0.513	0.960
**Insulin T0**	0.497	0.674	0.977	0.392	0.053
**HOMA index T0**	0.999	0.612	0.942	0.743	0.048
**Total cholesterol T0**	0.000	0.190	0.653	0.172	0.517
**LDL cholesterol T0**	0.000	0.424	0.736	0.373	0.720
**HDL cholesterol T0**	0.319	0.000	0.548	0.551	0.638
**Triglycerides T0**	0.731	0.003	0.000	0.106	0.003
**CRP T0**	0.110	0.273	0.465	0.170	0.017
**Cortisol T0**	0.050	0.058	0.021	0.066	0.000
**BAFF T0**	0.576	0.617	0.468	0.442	0.377
**PAF T0**	0.420	0.002	0.227	0.630	0.180
**TNF-α T0**	0.153	0.162	0.088	0.700	0.030
**MG T0**	0.491	0.964	0.821	0.683	0.400
**GA T0**	0.893	0.207	0.220	0.676	0.252
**glic/tot T0**	0.540	0.358	0.074	0.943	0.098
**Fasting blood glucose T12**	0.226	0.250	0.882	0.503	0.067
**Glycated hemoglobin T12**	0.323	0.030	0.098	0.982	0.557
**Insulin T12**	0.692	0.013	0.124	0.726	0.313
**HOMA index T12**	0.907	0.039	0.396	0.610	0.410
**Total cholesterol T12**	0.000	0.091	0.920	0.223	0.598
**LDL cholesterol T12**		0.273	0.531	0.363	0.329
**HDL cholesterol T12**	0.238		0.011	0.739	0.385
**Triglycerides T12**	-0.138	-0.498		0.535	0.019
**CRP T12**	0.199	-0.070	0.130		0.726
**Cortisol T12**	0.213	0.182	-0.467	-0.074	
**BAFF T12**	0.117	-0.051	0.286	0.140	0.109
**PAF T12**	-0.297	-0.593	0.373	0.180	-0.261
**TNF-α T12**	0.293	-0.233	0.491	0.474	-0.308
**MG T12**	0.193	0.191	-0.070	-0.081	-0.080
**GA T12**	-0.096	0.266	0.064	0.332	-0.084
**glic/tot T12**	-0.179	0.270	0.215	0.323	-0.215

	BAFF T12	PAF T12	TNF-α T12	MG T12	GA T12
**Age**	0.822	0.659	0.441	0.571	0.182
**Pre-gestational weight**	0.175	0.899	0.277	0.742	0.136
**Pre-gestational BMI**	0.294	0.675	0.189	0.872	0.215
**Birth weight**	0.935	0.308	0.166	0.559	0.146
**Fasting blood glucose T0**	0.670	0.724	0.810	0.893	0.889
**Glycated hemoglobin T0**	0.949	0.020	0.856	0.513	0.960
**Insulin T0**	0.497	0.674	0.977	0.392	0.053
**HOMA index T0**	0.999	0.612	0.942	0.743	0.048
**Total cholesterol T0**	0.000	0.190	0.653	0.172	0.517
**LDL cholesterol T0**	0.000	0.424	0.736	0.373	0.720
**HDL cholesterol T0**	0.319	0.000	0.548	0.551	0.638
**Triglycerides T0**	0.731	0.003	0.000	0.106	0.003
**CRP T0**	0.110	0.273	0.465	0.170	0.017
**Cortisol T0**	0.050	0.058	0.021	0.066	0.000
**BAFF T0**	0.576	0.617	0.468	0.442	0.377
**PAF T0**	0.420	0.002	0.227	0.630	0.180
**TNF-α T0**	0.153	0.162	0.088	0.700	0.030
**MG T0**	0.491	0.964	0.821	0.683	0.400
**GA T0**	0.893	0.207	0.220	0.676	0.252
**glic/tot T0**	0.540	0.358	0.074	0.943	0.098
**Fasting blood glucose T12**	0.226	0.250	0.882	0.503	0.067
**Glycated hemoglobin T12**	0.323	0.030	0.098	0.982	0.557
**Insulin T12**	0.692	0.013	0.124	0.726	0.313
**HOMA index T12**	0.907	0.039	0.396	0.610	0.410
**Total cholesterol T12**	0.000	0.091	0.920	0.223	0.598
**LDL cholesterol T12**		0.273	0.531	0.363	0.329
**HDL cholesterol T12**	0.238		0.011	0.739	0.385
**Triglycerides T12**	-0.138	-0.498		0.535	0.019
**CRP T12**	0.199	-0.070	0.130		0.726
**Cortisol T12**	0.213	0.182	-0.467	-0.074	
**BAFF T12**	0.117	-0.051	0.286	0.140	0.109
**PAF T12**	-0.297	-0.593	0.373	0.180	-0.261
**TNF-α T12**	0.293	-0.233	0.491	0.474	-0.308
**MG T12**	0.193	0.191	-0.070	-0.081	-0.080
**GA T12**	-0.096	0.266	0.064	0.332	-0.084
**glic/tot T12**	-0.179	0.270	0.215	0.323	-0.215

	glic/tot T12
**Age**	0.338
**Pre-gestational weight**	0.029
**Pre-gestational BMI**	0.284
**Birth weight**	0.074
**Fasting blood glucose T0**	0.437
**Glycated hemoglobin T0**	0.014
**Insulin T0**	0.345
**HOMA index T0**	0.530
**Total cholesterol T0**	0.528
**LDL cholesterol T0**	0.929
**HDL cholesterol T0**	0.016
**Triglycerides T0**	0.429
**CRP T0**	0.892
**Cortisol T0**	0.658
**BAFF T0**	0.477
**PAF T0**	0.942
**TNF-α T0**	0.043
**MG T0**	0.546
**GA T0**	0.013
**glic/tot T0**	0.001
**Fasting blood glucose T12**	0.508
**Glycated hemoglobin T12**	0.019
**Insulin T12**	0.126
**HOMA index T12**	0.429
**Total cholesterol T12**	0.897
**LDL cholesterol T12**	0.413
**HDL cholesterol T12**	0.192
**Triglycerides T12**	0.303
**CRP T12**	0.115
**Cortisol T12**	0.302
**BAFF T12**	0.562
**PAF T12**	0.409
**TNF-α T12**	0.794
**MG T12**	0.295
**GA T12**	0.000
**glic/tot T12**	

In the upper part of the table, we reported p-value, while in the lower part of the table, we reported R-value. HOMA index, homeostatic model assessment index; HDL cholesterol, high-density lipoprotein cholesterol; LDL cholesterol, low-density lipoprotein cholesterol; CRP, C-reactive protein.
